# Liver lymphatic drainage patterns follow segmental anatomy in a murine model

**DOI:** 10.1038/s41598-020-78727-y

**Published:** 2020-12-11

**Authors:** Nicola C. Frenkel, Susanna Poghosyan, André Verheem, Timothy P. Padera, Inne H. M. Borel Rinkes, Onno Kranenburg, Jeroen Hagendoorn

**Affiliations:** 1 Laboratory for Translational Oncology, Cancer Center, University Medical Center Utrecht and Utrecht University, Heidelberglaan 100, 3584CX Utrecht, The Netherlands; 2 E.L. Steele Laboratory for Tumor Biology, Department of Radiation Oncology, Massachusetts General Hospital and Harvard Medical School, Boston, MA USA

**Keywords:** Gastrointestinal system, Physiology, Gastrointestinal models, Gastrointestinal system, Hepatology, Lymphangiogenesis

## Abstract

The liver’s cellular functions are sustained by a hierarchical, segmentally-organized vascular system. Additionally, liver lymphatic vessels are thought to drain to perihepatic lymph nodes. Surprisingly, while recent findings highlight the importance of organ-specific lymphatics, the functional anatomy of liver lymphatics has not been mapped out. In literature, no segmental or preferential lymphatic drainage patterns are known to exist. We employ a novel murine model of liver lymphangiography and in vivo microscopy to delineate the lymphatic drainage patterns of individual liver lobes. Our data from blue dye liver lymphangiography show preferential lymphatic drainage patterns: Right lobe mainly to hepatoduodenal ligament lymph node 1 (LN1); left lobe to hepatoduodenal ligament LN1 + LN2 concurrently; median lobe showed a more variable LN1/LN2 drainage pattern with increased (sometimes exclusive) mediastinal thoracic lymph node involvement, indicating that part of the liver can drain directly to the mediastinum. Upon ferritin lymphangiography, we observed no functional communication between the lobar lymphatics. Altogether, these results show the existence of preferential lymphatic drainage patterns in the murine liver. Moreover, this drainage can occur directly to mediastinal lymph nodes and there is no interlobar lymphatic flow. Collectively, these data provide the first direct evidence that liver lymphatic drainage patterns follow segmental anatomy.

## Introduction

The complex and vital functions of the liver are sustained by central inflow of blood through a highly hierarchical, segmentally organized network of arteries and portal veins, with outflow through hepatic veins. The liver interstitial fluid is drained by lymphatic vessels and is estimated to produce 25–50% of the lymph in the thoracic duct^1–3^. Lymphatic vessels display increased density in cirrhosis and around liver tumors^4–6^. The liver lymphatic system is also involved in cancer, as both primary and secondary liver tumors often present with lymph node metastases in the hepatic pedicle^7,8^. Surprisingly, while the structure and function of organotypic lymphatic systems of the intestine, heart, skin, and brain meninges have recently been described^9–15^, the liver lymphatic system remains elusive. Micro-anatomical data from scanning electron microscopy studies have suggested that lymph originates from plasma filtered into the Space of Disse from the sinusoids, then flows via the Space of Mall to lymphatic capillaries near the portal triad^1,16,17^. From there, it is suggested that collecting lymphatics drain to hilar nodes. In addition, it is thought that there are lymphatic connections from the area around the central veins upwards along the suprahepatic inferior vena cava to the mediastinum^1,17^. Lastly, the liver capsule has been suggested to contain lymphatic vessels^1,18^. It remains unknown, however, whether lymphatic drainage from the liver to the perihepatic lymph nodes (i.e., the lymph nodes in the hepatoduodenal ligament, hepatogastric ligament, and around the suprahepatic inferior vena cava) occurs randomly or adheres to segmental anatomy. Here, we employ a novel technique for liver lymphangiography and in vivo microscopy in mice to delineate the pattern of drainage of the different liver lobes. We show that the liver lobes display preferential drainage patterns to specific lymph nodes and that there is no functional lymphatic communication between the liver lobes, providing the first direct evidence that liver lymphatic drainage follows segmental anatomy in a murine model.

## Results

### Intrahepatic lymphatic vessels are exclusively situated near portal triads

**Figure 1:**
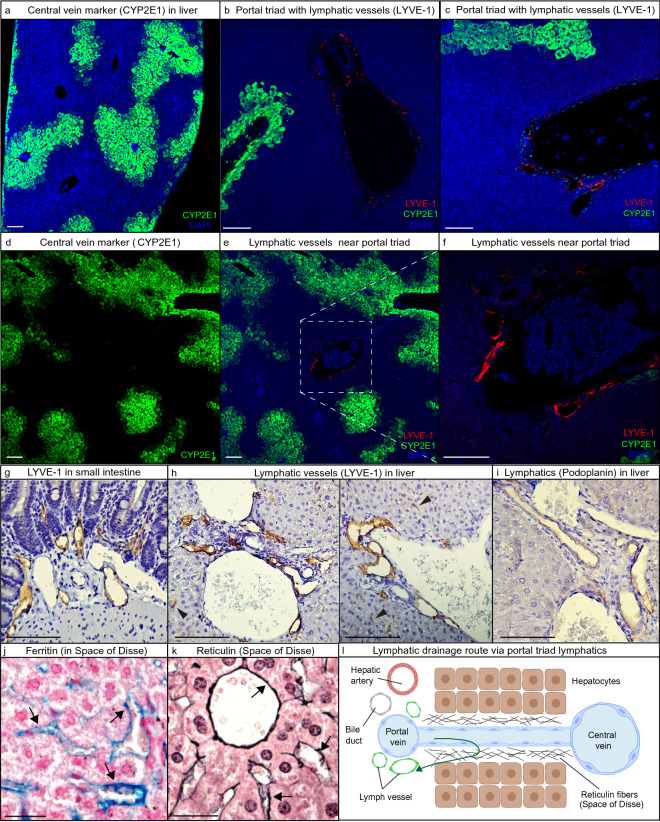
Lymphatic vessels in murine liver exclusively situated in the portal triad interstitium. Fluorescent Multiplex Immunohistochemistry (mIHC) with Tyramide Signal Amplification was used to stain for lymphatic vessels (LYVE-1) in the liver. (**a**) CYP2E1 is expressed by hepatocytes in close proximity to central veins and was used to distinguish portal triad interstitium from central vein interstitium. Scale bar = 100 µm. (**b**,**c**) Lymphatic vessels (LYVE-1) shown near portal triads. Scale bar = 50 µm. (**d**–**f**) Lymphatic vessels (LYVE-1) appear exclusively located at the portal triad as no lymphatics are seen near central veins (CYP2E1). Scale bar = 50 µm. (**g**–**i**) Using LYVE-1 as well as podoplanin to identify lymphatics, similar lymphatic vessels were observed. Arrowheads indicating LYVE-1 also lightly staining sinusoid endothelium^5^. Scale bar = 50 µm. (**j**,**k**) Representative images of IHC stainings for Perl’s Prussian blue (ferritin) and reticulin fiber (situated in the Space of Disse) suggest ferritin accumulation in the Space of Disse (arrows). Scale bar = 20 µm. (**l**) Schematic overview of lymphatic drainage in liver: from the sinusoids, to the Space of Disse, to lymphatic vessels near the portal triad.

The location of lymphatic vessels in the liver parenchyma has previously been described to be situated near the vessels of the portal triad^6,17^. To confirm these findings, we used fluorescent multiplex immunohistochemistry (mIHC) to explore the histological location of lymphatics in the liver parenchyma. We observed a clear discrimination of lymphatic vessels, using Lymphatic Vessel Endothelial Hyaluronan Receptor 1 (LYVE-1), with the small intestine lacteals and lymphatics serving as positive control tissue (Supplementary Fig. S1a). Standard IHC staining was performed using LYVE-1 as well as podoplanin as an additional lymphatic marker (Fig. 1g–i). LYVE-1 is known to also be expressed by sinusoid endothelium^5^ as can be seen in Fig. 1h (arrowheads). Using podoplanin as additional control and dilution of LYVE-1 allowed for specifically visualizing intensely stained lymphatic vessels versus light, occasional sinusoidal LYVE-1 staining. Using Cytochrome P450 Family 2 Subfamily E Member 1 (CYP2E1) as a marker of hepatocytes located near central veins, lymphatic vessels were observed exclusively in the vicinity of portal triads (Fig. 1a–f). Infused ferritin was observed in the space along the sinusoidal lining, corresponding with reticulin fibers situated in the Space of Disse. This would suggest ferritin flows via the Space of Disse and the portal triad, as has been previously suggested^17^. (Fig. 1l) We did not observe ferritin in the Space of Mall.

### Lymphangiography and in vivo microscopy of liver lymphatic drainage

**Figure 2:**
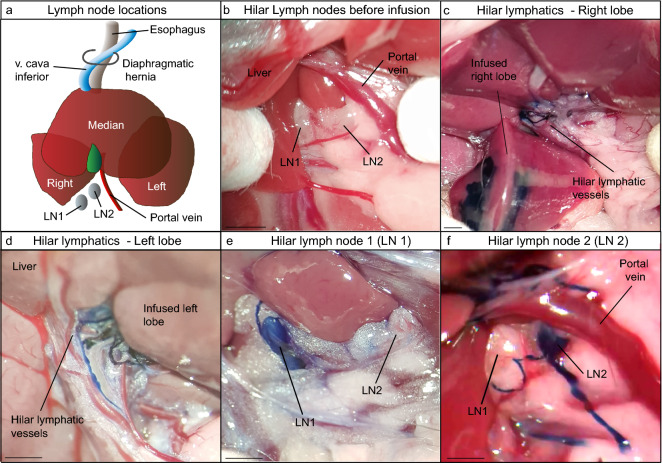
Murine liver lymphangiography model identifying hilar lymphatic vessels and lymph nodes. All C57Bl/6 mice (n = 57) had 2 hilar lymph nodes situated in the hepatoduodenal ligament. (**a**) Schematic overview of hilar lymph nodes (LN1 and LN2). (**b**) Representative image of hilar lymph nodes. (**c**,**d**) Liver lymphangiography was performed with 10% Evan’s blue dye into right lobe (n = 21), left lobe (n = 19) and median lobe (n = 17). Representative images of dye accumulating in hilar lymphatic vessels which can be seen to drain from the infused lobe towards the hilar region. (**e**,**f**) Representative images of dye accumulation in hilar LNs. All scale bars = 2 mm.

In all C57Bl/6 mice (n = 57), two hilar lymph nodes (LN1 and LN2) located in the hepatoduodenal ligament were observed (Fig. 2a,b). In the current literature, no segmental drainage or preferential lymphatic drainage patterns are known to exist in the liver^1,17^. Therefore, we performed liver lymphangiography with Evan’s blue dye, which is specifically taken up by lymphatics due to its molecular weight. Lymphangiography with Evan’s blue dye and in vivo microscopy showed visible dye accumulation in hilar lymphatic vessels and lymph nodes (Fig. 2c–f). These observations confirm that blue dye can be used to assess lymphatic drainage of the liver in vivo.

### Preferential drainage patterns per liver lobe

**Figure 3:**
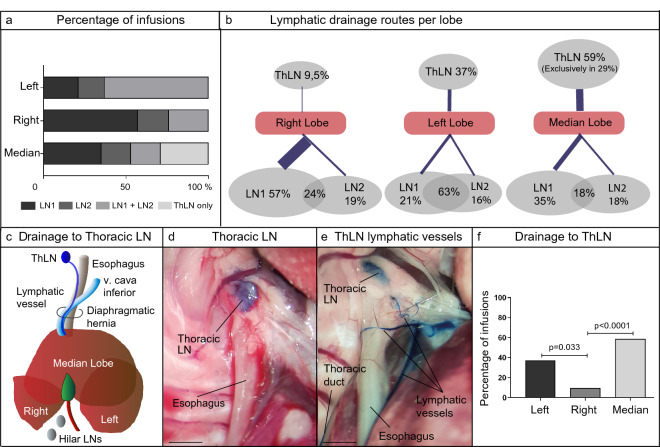
In vivo lymphangiography shows preferential drainage patterns per lobe. (**a**) Bar graph of Evan’s blue dye lobar drainage patterns (data shown are percentages). (**b**) Schematic overview of observed Evan’s blue dye drainage to LN1, LN2, combination LN1 + LN2 and ThLN per lobe. (**c**) Schematic overview of lymphatic drainage to ThLN, supplied by lymphatics running along the inferior vena cava and the esophagus. (**d**) Representative image of dye accumulation in ThLN. Scale bar = 2 mm. (**e**) Representative image of the direct lymphatic connection from the liver, running along the esophagus. This direct lymphatic route was observed in all blue-stained ThLN (total n = 19). Scale bar = 2 mm. (**f**) Bar graph of Evan’s blue dye drainage to ThLN per lobe (data shown are percentages). For the analysis of the Evan’s blue dye lymphangiographies, a Generalized Estimation Equation (GEE) model was used. For this model, it was notated per unit (per mouse) where the infusion site was (Left, Right or Median lobe) and at which location blue color dye was observed (LN1, LN2, ThLN). The data itself was notated categorically: either blue dye was observed (designated “1”) or not observed (designated “0”). Pairwise comparisons (Wald-Chi square tests) were performed for all-level combinations. Figure represents the pairwise comparison for lobe infusions and the concomitant positive coloring of ThLN. The infused lobe with the lowest percentage of positive ThLNs was used as reference point for the comparisons.

To assess whether different liver lobes have preferential drainage patterns, hilar lymph node coloring by Evan’s blue dye was tracked per injected lobe (Table 1). The right lobe’s main drainage route was via lymph node 1 (LN1, 57% vs LN2, 19%, p = 0.03). Left lobe infusion mostly showed concurrent drainage to LN1 and LN2 (63% to both LN1 + LN2, with only 21% to LN1 and 16% to LN2, resp.) with no significant difference between LN1 and LN2 (p = 0.70). For the median lobe, no preferential difference was found for LN1 or LN2 or their combination (35% vs 18% vs 18%, resp., p = 0.303) (Fig. 3a,b).

**Table 1:** Overview of lymphatic drainage patterns per lobe.

Lymphatic drainage pattern (Evan’s blue dye)	No. of mice (%)
**Left lobe**	19
Only LN1	4 (21.1)
Only LN2	3 (15.8)
LN1 and LN2	12 (63.2)
Thoracic LN	7 (36.8)
Only thoracic LN	0 (0)
**Right lobe**	21
Only LN1	12 (57.1)
Only LN2	4 (19)
LN1 and LN2	5 (23.8)
Thoracic LN	2 (9.5)
Only thoracic LN	0 (0)
**Median lobe**	17
Only LN1	6 (35.3)
Only LN2	3 (17.6)
LN1 and LN2	3 (17.6)
Thoracic LN	10 (58.8)
Only thoracic LN	5 (29.4)

### Thoracic lymph node: separate drainage route from the liver

Lymph fluid that passed the hilar lymph nodes (either LN1, LN2 or both) subsequently drained into the thoracic duct. Frequently, a blue stained lymph node in the superior mediastinum was also observed (Fig. 3c–e). This thoracic lymph node (ThLN) was always supplied by a vessel or pair of vessels running *directly* from the cranial side of the liver (i.e., not via the thoracic duct), alongside the suprahepatic inferior vena cava and the esophagus. These supplying lymphatics were observed every time ThLN stained blue (Fig. 2e). Notably, a different ThLN drainage pattern was observed for each lobe. The right lobe showed ThLN drainage in only 9.5% of the mice, while for the left lobe this was 37% (p = 0.03). Even more pronounced, the median lobe drained to ThLN in 59% of the mice (p = 0.00). Furthermore, unique to median lobe infusions, in 29% of the mice (p = 0.00) drainage to ThLN was seen without dye accumulation in LN1 or LN2 (Fig. 2a,b,f). Therefore, ThLN appears to offer the major (and in part of cases, only) drainage route for the median lobe.

### Absence of functional lymphatic communication between liver lobes

**Figure 4:**
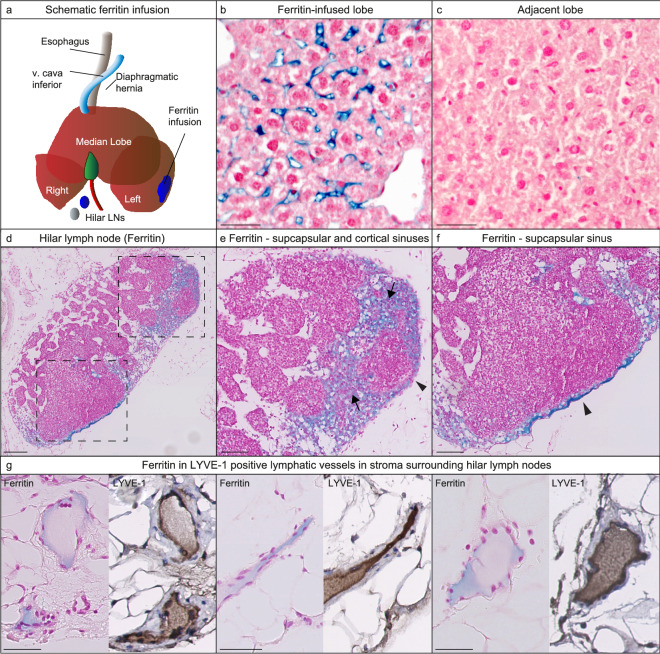
In vivo ferritin lymphangiography shows no interlobar lymphatic communication and confirms parenchymal drainage to hilar lymph nodes. (**a–c**) Ferritin stained with Perl’s Prussian Blue is visible in the infused lobes, but not in any the parallel lobes. 5 mice per liver lobe (n = 15 total) were infused with ferritin. Scale bar = 20 μm. (**d**) A mixture of ferritin and Evan’s blue dye was infused into the liver parenchyma. After lymph nodes were visually blue, they were harvested. Perl’s staining shows the presence of ferritin within the hilar lymph nodes, (**e**,**f**) specifically in the subcapsular (arrowheads) and cortical (arrows) sinuses. (**d**) Scale bar = 200 μm and (**e**,**f**) scale bar = 100 μm (**g**) Ferritin was also observed in the LYVE-1 positive lymphatic vessels surrounding the hilar lymph nodes. Scale bar = 50 μm.

Ferritin visualized by Perl’s Prussian blue staining was visible in the volumetrically infused lobes, but not in any of the adjacent liver lobes (Fig. 4a–c), thus showing that there are no functional lymphatic connections between liver lobes. This supports a segmental division of lymphatic vessels in the liver. Infusing the liver parenchyma with a mixture of Evan’s blue and ferritin allowed for the lymph nodes that visually turned blue to be investigated histologically for ferritin. Figure 4d–f shows ferritin in the hilar lymph node at the subcapsular and cortical sinuses after in vivo liver lymphangiography. Furthermore, ferritin was observed in the LYVE-1 positive lymphatic vessels surrounding the lymph nodes (Fig. 4g). These data confirm the lymphatic drainage of the liver parenchyma to the hilar lymph nodes.

## Discussion

Whereas the existence of lymphatic vessels in the liver and increased lymphatic density in several pathological processes have been shown, their functional anatomy has not been established^1,5,16,17^. Our data suggests that tracer dye drains to the Space of Disse and that vessels staining for lymphatic markers in the liver parenchyma are exclusively situated in the vicinity of portal triads, in accordance with current literature^6,17^. From the liver parenchyma, tracer dye drained directly to hilar and thoracic lymph nodes. However, the exact flow from ‘initial’ to ‘collecting’ lymphatics as well as the latter’s specific location remains to be elucidated.

Moreover, our results provide the first evidence that liver lymphatics are organized segmentally, in a murine model. It has been assumed that the liver lymphatic system contains a ‘deep’ (parenchymal) and ‘superficial’ (capsular) system without evidence for a segmental delineation, but this has been based mainly on early observational anatomical studies without modern IHC specific for lymphatic endothelial cells or intravital observation of functional lymphatics^1,17, 19–22^. A putative capsular lymphatic system could not be assessed in our study, as the liver capsule in mice is too thin and fragile to allow for separate tracer injection, but the ‘deep’ lymphatics that drain the larger volume of liver parenchyma could be mapped out. Since vascular lobar architecture of the mouse liver resembles the human segmental liver anatomy and its highly hierarchical organization of arteries, veins and bile ducts^23,24^, the data strongly suggest that the principle findings apply equally in man, although we suggest that further human studies on liver lymphatic drainage are mandated to confirm and expand our findings. Drainage of ‘lymphatic fluid’ from the liver has been observed in canine and porcine models, as well as in man, using various injection methods, tracers, and direct or radiological imaging to observe putative lymphatic vessels. However, segmental delineation was not studied^25–28^. In addition, it has been attempted to apply liver lymphangiography to identify ‘sentinel lymph nodes’ of the liver^25,27,28^. It remains unclear, however, whether the surgical concept of a sentinel lymph node applies to liver malignancies. Nevertheless, our data suggesting a segmental delineation of liver lymphatics may support further investigation into the existence of hepatic sentinel lymph nodes. In our study, we chose to infuse both Evan’s blue dye and ferritin at the same locations per lobe to ensure reproducibility. This location per lobe was chosen to be situated at the edge of the lobe furthest away from the hilar region where lymphatic flow exits the lobe (Supplementary Fig. S1b). This would ensure that the infused ferritin or dye had to traverse the entirety of the lobe before exiting. While this approach aided reproducibility in the experiment, a limitation is that we could not investigate whether regions *within* a lobe would also have different drainage patterns. For further studies in mice and in man, this would be a worthwhile consideration.

Mainly based on early work of Rouvière, it is believed that (part of) the liver lymphatics may drain directly to lymph nodes in the mediastinum^17,21,22^. In human anatomy, lymphatic vessels running along the hepatic veins and suprahepatically along the inferior vena cava are thought to drain towards mediastinal lymph nodes^17^. In contrast, a study examining fluorescent tracers to identify putative sentinel lymph nodes of the liver in pigs did not identify lymphatic pathways towards the diaphragm^27^. Our observation of drainage to thoracic lymph nodes supplied by lymphatic vessels running through the diaphragmatic hernia confirm direct liver drainage to the mediastinal compartment. Moreover, the preferential drainage pattern for the thoracic lymph node by the median lobe, suggest that part of the liver parenchyma preferentially drains to thoracic lymph nodes.

Clinically, these findings may have broad implications in surgical and oncological treatment. Surgically, extrahepatic metastatic spread, especially to perihepatic lymph nodes, is associated with decreased survival in both primary and secondary liver malignancies^7,8,29^. However, locoregional lymphatic spread is not a contraindication for surgery. Therefore, the finding that the liver can drain directly to mediastinal lymph nodes can potentially expand indications for local treatment. Also, delineation of preferential lymphatic drainage routes would aid decision making in terms of regional lymphadenectomy during partial hepatectomy for liver tumors. Oncologically, we speculate that further studies may elucidate whether patients with primary or secondary liver tumors and lymph node metastases confined to the liver-draining nodes that are not eligible for surgery may still benefit from local or regional treatment combined with systemic or targeted therapy that affects lymphatic dissemination.

## Methods

### Animals

Healthy male C57Bl/6 mice, ordered from the Jackson Laboratory, age 10–12 weeks and weight 25–30 g, were housed in groups of maximum 5 animals per cage in Type II cages with filter top at room temperature. The animals were kept under 12 h light/dark cycles and received standard chow pellets and water ad libitum. This research was approved by the Competent Authority, The Netherlands (Licence number AVD115002016614), which is advised by the Animal Ethics Committee. All protocols and experiments were then reviewed and approved by the Animal Welfare Body and were performed in accordance to the Dutch Law on Animal Experiments and the European Directive 2010/63/EU.

### Evan’s blue dye and ferritin lymphangiography

A dedicated mouse surgery room was used for all liver lymphangiographies. All operations were performed during the afternoon. Anesthesia was started by isoflurane vaporizer and induction chamber at 4–5% isofluorane. Afterwards, a face mask was used at 1–2% for maintaining anesthesia. Mice were also given Temgesic by subcutaneous injection preoperatively at 0.05 mg/kg. Median laparotomy was performed and the liver was exposed. For in vivo microscopy, lymphangiography was performed using Evan’s blue (Sigma, E2129), which is selectively taken up by lymphatic vessels due to its molecular weight, as described previously^30^. For liver lymphangiography, a 10 ml syringe, a 30 Gauge needle, a 75 cm infusion line and an infusion pump were used. After flushing with phosphate-buffered saline (PBS), the line and needle were filled with 10% Evan’s blue in PBS. The needle was gently inserted deep into the liver parenchyma and the pump was set at an infusion rate of 2 ml/h. After 50 s (total infusion volume: 28 μl) the needle was carefully removed and any residual bleeding was stemmed using cotton swabs. Per cage, the animals were assigned randomly to left lobe, median lobe or right lobe infusion. Only one infusion was performed per mouse. The infusion location could not be blinded. For the exact location of the insertion of the needle, we investigated different approaches during our pilot experiments. We believed injecting at the edge of the lobe furthest away from the hilar region would ensure that the dye had to flow through the entirety of the lobe before exiting the lobe. Moreover, to ensure reproducibility we kept the infusion location the same per lobe. As there is no previous data on liver lymphangiographies in mice, a sample number calculation could not be performed. We estimated around 25 animals per lobe were needed to assess the drainage patterns for the different lobes (total n of 75 mice). The primary outcome was the visual accumulation of blue coloring in lymphatic vessels and lymph nodes. If inadvertently inserted into a major vein or bile duct, within seconds blue dye was seen in veins or the common bile duct resulting in the animal’s withdrawal from the study (n = 18 of total 75 mice). In total, 57 out of 75 mice were included in the analysis. Blue dye accumulation in lymph vessels and lymph nodes was observed using a Leica M651 stereomicroscope, and the color intensity was graded 0–4. Only intensity 2 or higher were considered positive. Supplemental Figure S1c shows a schematic overview as well as example images of this grading system. After infusion, allowing 5–10 min to inspect all hilar lymph nodes for color patterns, a thoracotomy was performed and the thoracic duct and thoracic lymph node(s) were inspected. For histological identification of liver lymphatics, ferritin lymphangiography was used as described before for use in the mouse tail^31^. Ferritin liver lymphangiography was performed as described above for Evan’s blue liver lymphangiography. A 2 ml syringe was filled with ferritin solution (Sigma, F4503) and infused at 2 ml/h for 50 s. Subsequently, mice were sacrificed by perfusion-fixation and the liver was harvested for immunohistochemistry. 5 mice per liver lobe (n = 15 total) were infused with ferritin. For 3 mice the infusions had to be excluded from the experiment: For one mouse because the infusion had unfortunately disrupted a larger blood vessel and for 2 mice because no Prussian blue staining was observed in the infused lobe. Moreover, to investigate the hilar lymph nodes and the surrounding lymphatic vessels, we infused a mixture of ferritin with Evan’s blue dye into the parenchyma. The mixture consisted of 5 ml ferritin suspension with 200 ul of 10% Evan’s blue dye in PBS. This mixture allowed visualization of the draining lymph nodes that could then be harvested and stained with Perls’ Prussian blue to assess the drainage of ferritin.

### Immunohistochemistry and Perls’ Prussian Blue staining

Tissue was fixed in 4% buffered paraformaldehyde, paraffin-embedded and sections of 4 µm thickness were made. Sections were stained with Prussian Blue (Agilent Dako, AR15811-2), anti-reticulin (Agilent Dako, AR17911-2), anti-LYVE-1 (Abcam, ab14917, 1:200) and anti-Podoplanin (ThermoFisher, MA5-16113, 1:100). Images were taken at × 20 and × 40 magnification using a Nikon Eclipse E800 microscope.

### Fluorescent multiplex immunohistochemistry (mIHC) with tyramide signal amplification

The Tyramide SuperBoost Kit AF488 (ThermoFisher, B40922) was used. Paraffin-embedded sections of 4 µm thickness were deparaffinated and hydrated using a Xylene and ethanol range. The sections were then washed 2× for 5 min with demineralized water (demi-H_2_O) and boiled in citrate buffer (pH 6.0) for 20 min for antigen unmasking. The sections were left to cool down to room temperature (RT) and washed 3× for 5 min with demi-H_2_O. Endogenous peroxidase activity was quenched using 3% Hydrogen Peroxide for 30 min at RT. The sections were washed 2× for 5 min with 1× PBS and incubated with blocking buffer for 30 min at RT. The sections were then incubated with the primary antibody overnight at 4 °C. Afterwards, the sections were washed 3× for 10 min with 1× PBS and incubated for 60 min at RT with the poly-horseradish peroxidase (HRP)-conjugated secondary antibody. After washing 3× for 10 min with 1× PBS, the sections were incubated with the kit’s tyramide working solution for 10 min at RT. The working solution was washed away 3× for 10 min with 1× PBS. For the use of more than one antibody, the sections were then again placed in citrate buffer for renewed antigen unmasking. Afterwards, quenching and staining was performed as stated above, but a different Alexa-conjugated tyramide was used per antibody. Antibodies used were anti-LYVE-1 (Abcam, ab14917, 1:50), DAPI (4′,6-diamidino-2-phenylindole, Sigma, D9542, 1:1000) and CYP2E1 (Atlas antibodies, HPA009128, 1:50). Images were taken using a Zeiss LSM700 confocal microscope and Zeiss ZEN software program.

### Statistical analysis

All statistical analyses were performed by SPSS 20 (IBM SPSS, Chicago, IL). For the analysis of the blue lymph node patterns (LN1, LN2 and ThLN), positive lymph nodes (intensity score 2 or higher) were designated as “1” namely positive for staining. Non-stained lymph nodes (intensity score 1 or zero) were designated “0”. Images were blinded to scoring researcher for lobe infusion site. In Supplementary Fig. S1c, a schematic overview as well as example images of this scoring approach is shown. Because multiple lymph nodes were assessed in one mouse (repeated measurements of the same variable within one unit), a Generalized Estimation Equation (GEE) model was used. For the GEE model it was notated per unit (per mouse) where the infusion site was (Left, Right or Median lobe) and at which location blue color dye was observed (LN1, LN2, ThLN). Also in the model, the data was notated categorically: either blue dye was observed (designated “1”) or not observed (designated “0”). For the GEE model an Exchangeable working correlation matrix structure was used. Pairwise comparisons were performed using Wald-Chi square tests, for all-level combinations. Represented in Fig. 3f is the pairwise comparison for infusions in different lobes and the concomitant positive coloring of ThLN, using the lobe with the lowest percentage of positive ThLNs as reference point for the comparisons. Ferritin lymphangiography to investigate flow between infused and adjacent lobes resulted in a stark dichotomy of the data: infused lobes were positive (“1”) for ferritin while none of the adjacent lobes showed any ferritin and were all designated negative (“0”). This resulted in the problem of a complete separation in logistic regression models, whereby a statistical model could not be fitted to the data. Instead, the data was described as observed.

## Supplementary Information

Supplementary Information.
