# Supplementary material for: Liver lymphatic drainage patterns follow segmental anatomy in a murine model
Source: Sci Rep. 2020 Dec 11;10:21808. doi: 10.1038/s41598-020-78727-y (PMC7732834; doi:10.1038/s41598-020-78727-y)
Supplement: Supplementary file 1 — Supplementary Information. [file 41598_2020_78727_MOESM1_ESM.pdf]

Liver lymphatic drainage patterns follow segmental anatomy in a murine model

Nicola C. Frenkel[1], Susanna Poghosyan[1], André Verheem [1], Timothy P. Padera[2], Inne H.M. Borel Rinkes[1], Onno Kranenburg[1], Jeroen Hagendoorn[1*]

[1] Laboratory for Translational Oncology, University Medical Center Utrecht and Utrecht University, Heidelberglaan 100, 3584CX Utrecht, The Netherlands.

[2] E.L. Steele Laboratory for Tumor Biology, Dept. of Radiation Oncology, Massachusetts General Hospital and Harvard Medical School, Boston, MA, United States.

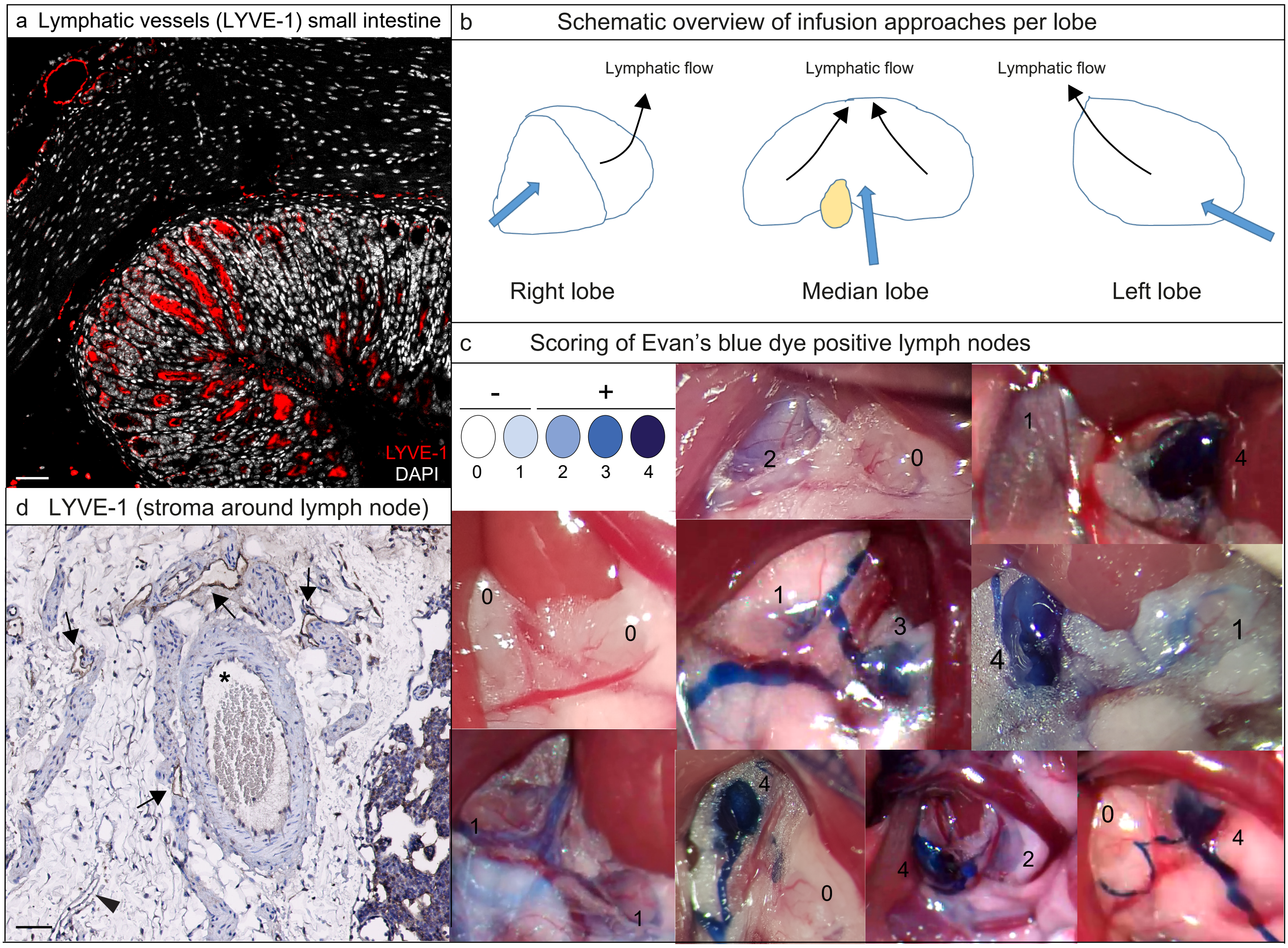

Supplementary Figure S1
(a) Fluorescent Multiplex Immunohistochemistry (mIHC) with Tyramide Signal Amplification was used to stain for lymphatic vessels (LYVE-1), small intestine lymphatics and lacteals were used as a positive control tissue for the staining of lymphatics in the liver parenchyma (Figure 1a-f). Scale bar = 50µm. (b) Schematic overview of the infusion locations per lobe (blue arrows). To ensure reproducibility the same location was used per lobe. The locations chosen were situated at the edge of the lobe opposite to where the lymphatic fluid exits the lobe at the hilar region (black arrows). This allows the infused solution to traverse the entirety of the lobe before reaching the hilar region. (c) A schematic overview illustrating the visual grading system of Evan's blue dye in the lymph nodes. A scale from 0-4 was used, increasing in intensity. Representative images showing examples of the color grading in vivo. (d) LYVE-1 staining used in the stroma surrounding the lymph node. LYVE-1 positive lymphatic vessels (arrows) as well as LYVE-1 negative arteries (*) and veins (arrowhead) can be observed in the stroma. Scale bar = 50µm.
